# Planctomycetes and macroalgae, a striking association

**DOI:** 10.3389/fmicb.2014.00267

**Published:** 2014-06-03

**Authors:** Olga M. Lage, Joana Bondoso

**Affiliations:** ^1^Department of Biology, Faculty of Sciences, University of PortoPorto, Portugal; ^2^CIMAR/CIIMAR – Interdisciplinary Centre for Marine and Environmental Research, University of PortoPorto, Portugal

**Keywords:** planctomycetes, macroalgae, biofilm, association, macroalgae exudates

## Abstract

Planctomycetes are part of the complex microbial biofilm community of a wide range of macroalgae. Recently, some studies began to unveil the great diversity of Planctomycetes present in this microenvironment and the interactions between the two organisms. Culture dependent and independent methods revealed the existence of a great number of species but, so far, only less than 10 species have been isolated. Planctomycetes comprise the genera *Rhodopirellula*, *Blastopirellula*, and *Planctomyces*, *Phycisphaera* and the uncultured class OM190 and some other taxa have only been found in this association. Several factors favor the colonization of macroalgal surfaces by planctomycetes. Many species possess holdfasts for attachment. The macroalgae secrete various sulfated polysaccharides that are the substrate for the abundant sulfatases produced by planctomycetes. Specificity between planctomycetes and macroalgae seem to exist which may be related to the chemical nature of the polysaccharides produced by each macroalga. Furthermore, the peptidoglycan-free cell wall of planctomycetes allows them to resist the action of several antimicrobial compounds produced by the macroalgae or other bacteria in the biofilm community that are effective against biofouling by other microorganisms. Despite the increase in our knowledge on the successful planctomycetes-macroalgae association, a great effort to fully understand this interaction is needed.

## Introduction

Planctomycetes are a peculiar group of bacteria within the *Planctomycetes*, *Verrucomicrobia*, *Chlamydiae* (PVC)—superphylum. They share with archaea or eukaryotes some distinctive characteristics such as peptidoglycan-less cell walls of proteic nature (Lage, 2013), a complex system of endomembranes forming a unique cell plan (Lage et al., 2013; Santarella-Mellwig et al., 2013), the presence of compartments like the anammoxosome (Van Teeseling et al., 2013), budding reproduction in many of their members (Ward et al., 2006) and the lack of the division protein FtsZ (Pilhofer et al., 2008), endocytosis (Lonhienne et al., 2010) and the presence of membrane coat (MC)—like proteins (Santarella-Mellwig et al., 2010). Some of these features place planctomycetes in the center of the discussion of the eukaryotic cell origin (Devos and Reynaud, 2010; Reynaud and Devos, 2011; Fuerst and Sagulenko, 2013).

Metabolically, planctomycetes are mainly aerobic, mesophilic, and neutrophilic organisms. A particular group of planctomycetes, the anaerobic ammonium oxidation (anammox) species, are strict anaerobes. Their diversified metabolism allows them to colonize a wide variety of ecosystems ranging from aquatic (marine, brackish, freshwater, sediments, and marine snow) to terrestrial habitats as well as several extreme environments such as desert soils (Abed et al., 2010; Andrew et al., 2012), hypersaline environments (Baumgartner et al., 2009; Schneider et al., 2013), hot springs (Tekere et al., 2011; Bohorquez et al., 2012), acidophilic habitats (Ivanova and Dedysh, 2012; Lucheta et al., 2013), glacial waters (Liu et al., 2006; Zeng et al., 2013) and Antarctic soils and waters (Newsham et al., 2010; Piquet et al., 2010), hydrocarbon polluted environments (Abed et al., 2011)and other polluted habitats (Reed et al., 2002; Chouari et al., 2003; Caracciolo et al., 2005; Akob et al., 2007; Halter et al., 2011). Furthermore, their association with a great number of diverse eukaryotic organisms has been reported. These include sponges (Webster et al., 2001; Pimentel-Elardo et al., 2003; Zhu et al., 2008; Costa et al., 2013), ascidians (Oliveira et al., 2013), corals (Yakimov et al., 2006; Webster and Bourne, 2007; Duque-Alarcón et al., 2012), prawns (Fuerst et al., 1997), macrophytes (Hempel et al., 2008) and lichens (Grube et al., 2012). They were also found in sphagnum peat bogs (Kulichevskaia et al., 2006), the rock below the lichens (Bjelland et al., 2011) and in the rizosphere of several plants (Jensen et al., 2007; Zhao et al., 2010; Zhang et al., 2013).

Recently, various studies showed that planctomycetes are widespread in the biofilm community of several species of macroalgae and present a high diversity (Bengtsson and Ovreas, 2010; Lachnit et al., 2011; Lage and Bondoso, 2011). Besides a fundamental role in the primary production, beds of macroalgae along ocean coastlines provide the needed structure complexity, habitat and food for a huge and variable community of organisms which range from microscopic forms to larger organisms like fishes. Macroalgae are the dominant habitat-forming organisms on temperate coastlines (Campbell et al., 2014) and offer shelter for many forms of life that can thus avoid predation by higher forms in the food chain. This is particularly evident in the large brown algal kelp forests. At a microscopic scale, macroalgal surfaces harbor a rich community composed by bacteria, fungi, diatoms, protozoa, spores and larvae of marine invertebrates (Lachnit et al., 2011) that can benefit from the availability of a range of organic carbon sources produced by algae (Armstrong et al., 2001). Bacteria are dominant among primary colonizers (Lachnit et al., 2009). The two major groups are *Bacteroidetes* and *Proteobacteria* followed by *Firmicutes*, *Actinobacteria*, *Verrucomicrobia*, and *Planctomycetes* (Goecke et al., 2013). In this review, we explore several aspects of the interaction between planctomycetes and macroalgae, a topic that recently started to be unveiled.

## Macroalgae that harbor planctomycetes

Planctomycetes are frequent colonizers of macroalgae from the three phyla, Chlorophyta (green algae), Rhodophyta (red algae) and Heterokontophyta (brown algae). Planctomycetes colonization was observed for ulvacean algae like *Cladophora* sp. (Yoon et al., 2014), *Ulva compressa* (Hengst et al., 2010), *Ulva intestinalis* (Hengst et al., 2010; Lachnit et al., 2011; Lage and Bondoso, 2011), *Ulva australis* (Longford et al., 2007; Burke et al., 2011), *Ulva prolifera* (Liu et al., 2010), and *Ulva* sp. (Lage and Bondoso, 2011; Bondoso et al., 2013). This group was also reported to be present in the green macroalgae *Chara aspera* (Hempel et al., 2008) and *Caulerpa taxifolia* (Meusnier et al., 2001). Epiphytic planctomycetes were also found in the red algae *Porphyra umbilicalis* (Miranda et al., 2013), *Laurencia dendroidea* (De Oliveira et al., 2012), *Delisea pulchra* (Longford et al., 2007), and *Gracilaria vermiculophylla* (Lachnit et al., 2011). Isolates were retrieved from *Chondrus crispus*, *Mastocarpus stellatus*, *Gracilaria bursa-pastoris*, *Gelidium pulchellum*, *Grateloupia turuturu*, and *Porphyra dioica* (Lage and Bondoso, 2011). The presence of planctomycetes on *Chondrus crispus*, *Mastocarpus stellatus*, and *Porphyra dioica* was detected by molecular methods (Bondoso et al., 2013). A novel order of planctomycetes containing one species isolated from *Porphyra* sp. was described (Fukunaga et al., 2009). 16S rRNA clone libraries from the brown algae *Fucus vesiculosus* revealed a great diversity of planctomycetes (Lachnit et al., 2011). Planctomycetes were isolated from other brown algae like *Fucus spiralis*, *Sargassum muticum*, *Laminaria* sp. (Lage and Bondoso, 2011), and *Laminaria hyperborea* (Bengtsson and Ovreas, 2010). The presence of planctomycetes has also been confirmed in *Fucus spiralis*, *Sargassum muticum* by Bondoso et al. (2013) and in *Saccharina latissima* and *L. digitata* (Bengtsson, unpublished results). These data suggest that planctomycetes are widespread among macroalgae which can be used for the discovery of novel planctomycetes species.

## Planctomycetes associated with macroalgae

Although the abundance of planctomycetes is usually observed to be low in marine environments (Rusch et al., 2007) and some macroalgae (Burke et al., 2011; Lachnit et al., 2011; Miranda et al., 2013), Bengtsson and Ovreas (2010) showed, by FISH, that planctomycetes are dominant on *Laminaria hyperborean* where they can account for up to 51–53% of the bacterial biofilm cells.

**Figure 1:**
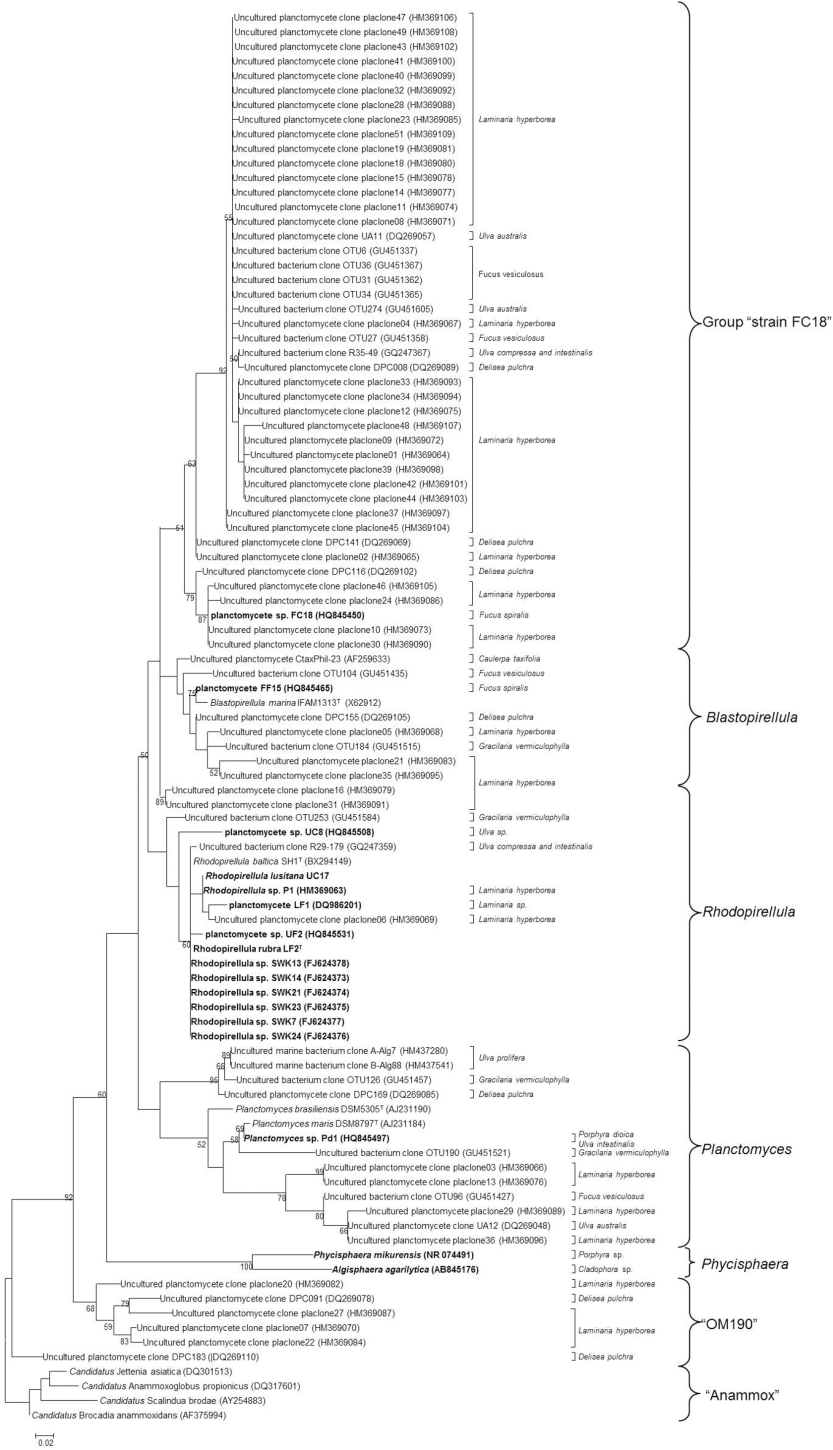
**Maximum-Likelihood tree of 16S rRNA gene sequences of planctomycetes associated with macroalgae downloaded from NCBI database**. The final set consisted of 116 sequences above 500 bp. Strains in bold represent the isolates from macroalgae described to date. The numbers beside nodes are the percentages for bootstrap analyses; only values above 50% are shown. Scale bar = 0.02 substitutions per 100 nucleotides. The different groups are presented on the right. Anammox 16S rRNA gene sequences were used as outgroup.

About 30% of all the studies on macroalgae bacterial communities report the presence of planctomycetes and almost 4% of sequences from these studies belong to the phylum *Planctomycetes* (Hollants et al., 2013). Planctomycete communities on macroalgae can be highly diverse varying from only one to 24 OTUs at a 97% cut-off in the 16S rRNA gene per macroalgae (Table 1). With the use of specific primers for planctomycetes, Bengtsson and Ovreas (2010) defined 16 OTUs associated with the kelp *Laminaria hyperborea*, each representing a different species and Bondoso et al. (2013), using PCR-DGGE, identified a total of 21 different OTUs associated with six macroalgae. In a pyrosequencing study, the red macroalga *Porphyra umbilicalis* was found to harbor 24 different OTUs belonging to planctomycetes (Miranda et al., 2013). In total, more than 60 potential different species of planctomycetes are associated with macroalgae and the majority were not isolated in pure culture (Figure 1). So far, only 10 species were isolated from macroalgae (Winkelmann and Harder, 2009; Bengtsson and Ovreas, 2010; Lage and Bondoso, 2011) on the basis of the 97% cut-off defined for species delineation (Stackebrandt, 2002) of which four were validly described (Fukunaga et al., 2009; Bondoso et al., 2014; Yoon et al., 2014). The communities of planctomycetes comprise mainly members related to the cultured genera *Blastopirellula*, *Rhodopirellula*, and *Planctomyces* (Figure 1) and to the class *Phycisphaerae* which contains the genera *Phycisphaera* and *Algisphaera*. The most abundant taxon reported in culture-independent studies is related to an isolate from *Fucus spiralis*, strain FC18 (Lage and Bondoso, 2011), and can be found in almost all the macroalgae studied but predominantly in the brown macroalgae *Fucus* sp. and *Laminaria hyperborea*. The uncultured class OM190 (SILVA taxonomy), a deeply branching group within the *Planctomycetes*, is also usually reported as being associated to macroalgae (Figure 1, Table 1).

**Table 1:** **Abundance and phylogenetic affiliation of planctomycetes associated with macroalgae**.

					**Planctomycetes**			
**Macroalgae**	**Species/genus**	**Location**	**Used method**	**Percentage**	**Number of genera^a^**	**Number of species^a^**	**Phylogenetically related to**	**References**
Chlorophyta (Green)	*Caulerpa taxifolia*	Philippines	16S rRNA gene libraries	ND	1	1	*Blastopirellula*	Meusnier et al., 2001
	*Chara aspera*	Lake Constance	Fluorescence in situ hybridization (FISH)	2-3	ND	ND	ND	Hempel et al., 2008
	*Cladophora sp*.	Sado Island, Japan	Isolation	ND	1	1	*Phycisphaera*	Yoon et al., 2014
	*Ulva australis*	Bare Island, Australia	16S rRNA gene libraries	2	2	2	*Blastopirellula, Planctomyces*	Longford et al., 2007
		Shark Point, Clovelly, Australia	16S rRNA gene libraries	3.4	ND	ND	ND	Burke et al., 2011
	*Ulva compressa* and *intestinalis*	Chañaral Bay, Chile	T-RFLP	1.3	1	1	*Rhodopirellula* and planctomycete FC18	Hengst et al., 2010
	*Ulva intestinalis*	Kiel fjord, Germany	16S rRNA gene libraries	ND	1	1	Planctomycete FC18	Lachnit et al., 2011
		Porto and Viana do Castelo, Portugal	Isolation	ND	1	1	*Planctomyces*	Lage and Bondoso, 2011
	*Ulva profilera*	Jiaozhou Bay, China	16S rRNA gene libraries	1	1	1	Planctomycete FC18	Liu et al., 2010
	*Ulva* sp.	Porto and Viana do Castelo, Portugal	Isolation	ND	2	5	*Rhodopirellula*	Lage and Bondoso, 2011
		Porto and Viana do Castelo, Portugal	DGGE	ND	1	2	Planctomycete FC18	Bondoso et al., 2013
Rhodophyt (Red)	*Chondrus crispus*	Porto and Viana do Castelo, Portugal	Isolation	ND	1	2	*Rhodopirellula*	Lage and Bondoso, 2011
		Porto and Viana do Castelo, Portugal	DGGE	ND	1	2	Planctomycete FC18	Bondoso et al., 2013
	*Delisea pulchra*	Bare Island, Australia	16S rRNA gene libraries	8	3	7	*Blastopirellula, Planctomyces*, Planctomycete FC18, OM190	Longford et al., 2007
	*Gelidium pulchellum*	Porto and Viana do Castelo, Portugal	Isolation	ND	1	1	*Rhodopirellula*	Lage and Bondoso, 2011
	*Gracilaria bursa-pastoris*	Porto and Viana do Castelo, Portugal	Isolation	ND	2	2	*Rhodopirellula*	Lage and Bondoso, 2011
	*Gracilaria vermiculophylla*	Kiel fjord, Germany	16S rRNA gene libraries	ND	3	6	*Blastopirellula, Rhodopirellula, Planctomyces*	Lachnit et al., 2011
	*Grateloupia turuturu*	Porto and Viana do Castelo, Portugal	Isolation	ND	1	1	*Rhodopirellula*	Lage and Bondoso, 2011
	*Laurencia dendroidea*	Búzios and Mangaratiba, Brasil	Transcriptome	ND	ND	ND	ND	De Oliveira et al., 2012
	*Mastocarpus stellatus*	Porto and Viana do Castelo, Portugal	Isolation	ND	1	2	*Rhodopirellula*	Lage and Bondoso, 2011
			DGGE	ND	2	3	*Rhodopirellula*, OM190	Bondoso et al., 2013
	*Porphyra dioica*	Porto and Viana do Castelo, Portugal	Isolation	ND	2	2	*Rhodopirellula, Planctomyces*	Lage and Bondoso, 2011
		Porto and Viana do Castelo, Portugal	DGGE	ND	2	2	*Rhodopirellula* and planctomycete FC18	Bondoso et al., 2013
	*Porphyra umbilicalis*	Schoodic Point, USA	Pyrosequencing	0.03–4.06	ND	24	*Planctomyces, Phycisphaera, Rhodopirellula*	Miranda et al., 2013
	*Porphyra* sp.	Mikura Island, Japan	Isolation	ND	1	1	*Phycisphaera*	Fukunaga et al., 2009
Heterokontophyt (Brown)	*Fucus spiralis*	Porto and Viana do Castelo, Portugal	Isolation	ND	2	6	*Rhodopirellula, FC18*	Lage and Bondoso, 2011
		Porto and Viana do Castelo, Portugal	DGGE	ND	1	1	Planctomycete FC18	Bondoso et al., 2013
	*Fucus vesiculosus*	Bare Island, Australia	16S rRNA gene libraries	ND	3	6	*Blastopirellula, Planctomyces*, Planctomycete FC18	Lachnit et al., 2011
	*Laminaria hyperborea*	Bergen, Norway	FISH and 16S rRNA gene libraries	23.7–52.5	4	16	*Blastopirellula, Planctomyces*, Planctomycete FC18, *Rhodopirellula*, OM190	Bengtsson and Ovreas, 2010
		Bergen, Norway	DGGE	46.3	5	8	*Blastopirellula, Planctomyces*, Planctomycete FC18, *Rhodopirellula*, OM190	Bengtsson et al., 2010
		Bergen, Norway	454-pyrosequencing	55.7	ND	ND	*Rhodopirellula*, Planctomycete FC18,	Bengtsson et al., 2012
	*Laminaria* sp.	Porto and Viana do Castelo, Portugal	Isolation	ND	1	1	*Rhodopirellula*	Lage and Bondoso, 2011
	*Sargassum muticum*	Porto and Viana do Castelo, Portugal	Isolation	ND	1	2	*Rhodopirellula*	Lage and Bondoso, 2011
		Porto and Viana do Castelo, Portugal	DGGE	ND	3	7	*Planctomyces*, Planctomycete FC18, *Rhodopirellula*	Bondoso et al., 2013

a *The sequences reported in the studies above were grouped using cd-hit-est (Huang et al., 2010) based on 97% (species) or 95% (genus) cut-off similarity in the 16S rRNA gene*.

*ND, not determined*.

The planctomycetes associated with red and brown macroalgae seem to have a higher diversity than the ones colonizing green algae (Figure 1 and Table 1). This finding was reported by Lachnit et al. (2011) where only one OTU was associated with *Ulva intestinalis*, but 7 and 6 OTUs were, respectively, associated with *Delisea pulchra* and *Fucus vesiculosus*.

The communities of planctomycetes comprise taxa that were never found before in other habitats, suggesting a specific association with the macroalgae. Thirty nine out of 116 total sequences present in databases were found to be limited to macroalgal surfaces. Moreover, the study performed by Bondoso et al. (2013) also suggested that this association is host-specific and does not change with the geographical location of the macroalgae.

## Interactions between macroalgae and planctomycetes

The dynamic marine environments where macroalgae live are affected by diverse biotic and abiotic factors which contribute to, and influence the microbial community of their biofilms. Fundamental for this biofilm formation is the complex chemistry of macroalgal surfaces composed of exudates of secondary metabolites and extracellular exopolymeric substances (EPS) (Goecke et al., 2013). The chemistry varies among macroalgal species making each species a unique microenvironment, which induces a unique microbial community. Planctomycetes should be able to adapt easily to these complex environments. They are highly responsive to changes in environmental conditions through complex adaptation machinery. This was observed in *Rhodopirellula baltica* under stress response to temperature and salinity (Wecker et al., 2009).

Macroalgae produce or release many molecules that can be rich sources of substrates for planctomycetes nutrition. Algal macromolecules include sulfated polysaccharides like carrageenan and agar from red algae, alginate, fucan and laminarinan from brown algae and cellulose and ulvan from green algae. Planctomycetes are well tailored for the utilization of sulfated polysaccharides as revealed by the analysis of the marine *R. baltica* SH1^T^ genome where the presence of 110 sulphatases was detected (Glockner et al., 2003). Furthermore, Wegner et al. (2013) also found an exceptionally high number of sulphatase genes in the recently sequenced genomes of nine *Rhodopirellula* strains. These authors also verified in *R. baltica* SH1^T^ sulphatase expression profiles in cells grown on different sulfated polysaccharides. Polysaccharide utilization was also confirmed by the work of Jeske et al. (2013) where *R. baltica* was checked for potential utilization of several polymers. It was able to utilize laminarin, mannitol, pectin, chondroitin sulfate, N-acetylgalactosamine, and D-glucuronic acid. Cellobiose, a product of cellulose degradation, could also be used as carbon source. Weak or moderate degradation was obtained for mannan and its monomer D-mannose, the disaccharide sucrose and D-xylose. The novel species *R. rubra* and *R. lusitana*, both isolated from macroalgae, were shown to utilize the majority of the monomers that constitute the main polysaccharides secreted by macroalgae such as fucose, galactose, xylose, rhamnose, and manitol (Bondoso et al., 2014). Agarolytic activity was also described in a novel representative of the class Phycisphaera, *Algisphaera agarilytica*, isolated from the marine alga *Cladophora* sp. (Yoon et al., 2014). As frequent inhabitants of phytodetrial macroaggregates in marine environments, planctomycetes mineralize organic matter, intervening in important transformations in the global carbon cycle in the sea (DeLong et al., 1993). Very recently, Erbilgin et al. (2014) provided evidence by a metabolic activity screen that Bacterial Microcompartments (BMCs) present in planctomycetes are involved in the degradation of a number of plant and algal cell wall sugars, namely L-fucose and L-rhamnose. This work further supports the great relevance of algal exudates on planctomycetes physiology especially for those associated with macroalgae.

The different substrates produced by each macroalga may explain the specificity of planctomycetes to the algal host. It was found that the same algal species from two localities demonstrated high similarities in the composition of associated planctomycetes (Bondoso et al., 2013). A core of evidence seems to point to the algal host as the main factor controlling the composition and structure of epiphytic bacterial communities (Wahl, 2008; Lachnit et al., 2009, 2011; Hengst et al., 2010). Comparable results were reported for the epibiotic bacterial communities living on corals which seemed to be determined by the nature and composition of host exudates due to strong seasonal effects (Guppy and Bythell, 2006).

Planctomycetes colonize macroalgal surfaces; an attached life style has been well recognized for these bacteria and when in pelagic environments they are mainly associated with particles like marine snow (DeLong et al., 1993). The presence of a holdfast of glycoproteic nature (Lage, 2013; Lage et al., 2013) favors attachment and, thus, the colonization of surfaces (Gade et al., 2005; Lage, 2013; Lage et al., 2013).

Another factor that favors the colonization of macroalgal biofilms by planctomycetes is their ability to resist several antibiotics. These can be produced by the macroalgae or by other competing bacteria in the biofilm. One of the methods to achieve planctomycetes isolation in culture is precisely based on this resistance to antibiotics (Schlesner, 1994; Winkelmann and Harder, 2009; Lage and Bondoso, 2011). Resistance to β-lactam antibiotics that affect peptidoglycan biosynthesis is due to the absence of this molecule in their cell wall.

In a study of the behavior of planctomycetes toward antibiotics, Cayrou et al. (2010) showed that five reference strains of planctomycetes were resistant to β-lactams, to the quinolone nalidixic acid and to the glycopeptide vancomycin. The organisms were, however sensitive to tetracycline and doxycycline. Most were also resistant to chloramphenicol and the aminoglycoside gentamicin as well as rifampicin. A variable resistance to the association sulfomethoxazole/trimethoprim was obtained.

The potential benefits of the planctomycetes to the macroalgae can only be hypothesized. Being heterotrophs, planctomycetes can mineralize organic molecules producing inorganic compounds that meet the nutritional needs of macroalgae. These may also profit from the production of growth factors or antimicrobial molecules by the planctomycetes. It has been shown that morphogenetic factors like thallusin, isolated from an epiphytic marine bacterium, are indispensable to the foliaceous morphology of macroalgae (Matsuo et al., 2005). Unknown factors may be due to planctomycetes. The production of bioactive molecules by planctomycetes was initially searched by genome mining in *R. baltica* (Donadio et al., 2007) and subsequently in 13 genomes (Jeske et al., 2013). Two small nonribosomal peptide synthetases (NRPSs), two monomeric polyketide synthases (PKSs), and a bimodular hybrid NRPS–PKS were found in the genome of *R. baltica* which are probably involved in the synthesis of five different, unknown bioactive products (Donadio et al., 2007). In the 13 genomes analyzed, 102 genes or gene clusters putatively related with the production of secondary metabolites like bacteriocin encoding genes, putative lantibiotic-encoding gene, ectoine synthesis gene cluster, putative phenazine encoding gene cluster were found (Jeske et al., 2013). The potential production of these bioactive molecules may help the macroalgae to control their colonization by undesired bacteria or fungi. Furthermore, we can hypothesize that bioactive molecules should be important for planctomycetes in the process of macroalgal colonization and posterior defense against competitors.

Bacteria can also impact negatively on macroalgae. Members of Bacteroidetes and Gammaproteobacteria can induce several diseases like “shot hole disease” or “hole-rotten disease” (Goecke et al., 2013). Up to now, pathogenicity from planctomycetes on macroalgae was never reported.

## Conclusions

As shown in this review, macroalgae are promising environments for in-depth study of planctomycetes. Macroalgae possess a relatively high number and a diverse community of planctomycetes in their biofilm. Thus, they represent great potential for the discovery of new taxa that can be isolated from these habitats and their characterization could provide new knowledge on the morphology, physiology and ecology of planctomycetes and on this interaction. New research on the structure, succession and dynamics of the community in this relationship, namely the relation with the macroalgal life cycle, the temporality of planctomycetes diversity and composition and their association with other macroalgae will give new highlights in the ecology of this interaction. Metabolomic approaches will allow obtaining insights into the mechanisms of the nutritional relationship and the role of planctomycetes in biofilm formation and maintenance. The increasing scientific interest in the biology of planctomycetes and biofilms will, most certainly, generate new exciting knowledge that will allow a better comprehension of this association.

### Conflict of interest statement

The authors declare that the research was conducted in the absence of any commercial or financial relationships that could be construed as a potential conflict of interest.

## References

[B1] Abed R. M. M. Al Kharusi S. Schramm A. Robinson M. D. (2010). Bacterial diversity, pigments and nitrogen fixation of biological desert crusts from the Sultanate of Oman. FEMS Microbiol. Ecol. 72, 418–428 10.1111/j.1574-6941.2010.00854.x 20298501

[B2] Abed R. M. M. Musat N. Musat F. Mußmann M. (2011). Structure of microbial communities and hydrocarbon-dependent sulfate reduction in the anoxic layer of a polluted microbial mat. Mar. Pollut. Bull. 62, 539–546 10.1016/j.marpolbul.2010.11.030 21194714

[B3] Akob D. M. Mills H. J. Kostka J. E. (2007). Metabolically active microbial communities in uranium-contaminated subsurface sediments. FEMS Microbiol. Ecol. 59, 95–107 10.1111/j.1574-6941.2006.00203.x 17233747

[B4] Andrew D. R. Fitak R. R. Munguia-Vega A. Racolta A. Martinson V. G. Dontsova K. (2012). Abiotic factors shape microbial diversity in Sonoran desert soils. Appl. Environ. Microbiol. 78, 7527–7537 10.1128/AEM.01459-12 22885757 PMC3485727

[B5] Armstrong E. Yan L. Boyd K. G. Wright P. C. Burgess J. G. (2001). The symbiotic role of marine microbes on living surfaces. Hydrobiologia 461, 37–40 10.1023/A:1012756913566

[B6] Baumgartner L. K. Dupraz C. Buckley D. H. Spear J. R. Pace N. R. Visscher P. T. (2009). Microbial species richness and metabolic activities in hypersaline microbial mats: insight into biosignature formation through lithification. Astrobiology 9, 861–874 10.1089/ast.2008.0329 19968463

[B7] Bengtsson M. M. Ovreas L. (2010). Planctomycetes dominate biofilms on surfaces of the kelp *Laminaria hyperborea*. BMC Microbiol. 10:261 10.1186/1471-2180-10-261 20950420 PMC2964680

[B8] Bengtsson M. M. Sjotun K. Lanzen A. Ovreas L. (2012). Bacterial diversity in relation to secondary production and succession on surfaces of the kelp *Laminaria hyperborea*. ISME J. 6, 2188–2198 10.1038/ismej.2012.67 22763650 PMC3505018

[B9] Bengtsson M. M. Sjotun K. Ovreås L. (2010). Seasonal dynamics of bacterial biofilms on the kelp *Laminaria hyperborea*. Aquat. Microb. Ecol. 60, 71–83 10.3354/ame01409

[B10] Bjelland T. Grube M. Hoem S. Jorgensen S. L. Daae F. L. Thorseth I. H. (2011). Microbial metacommunities in the lichen-rock habitat. Environ. Microbiol. Rep. 3, 434–442 10.1111/j.1758-2229.2010.00206.x 23761305

[B11] Bohorquez L. C. Delgado-Serrano L. Lopez G. Osorio-Forero C. Klepac-Ceraj V. Kolter R. (2012). In-depth characterization via complementing culture-independent approaches of the microbial community in an acidic hot spring of the Colombian Andes. Microb. Ecol. 63, 103–115 10.1007/s00248-011-9943-3 21947461

[B12] Bondoso J. Albuquerque L. Lobo-Da-Cunha A. Da Costa M. S. Harder J. Lage O. M. (2014). Rhodopirellula lusitana sp. nov. and Rhodopirellula rubra sp. nov., isolated from the surface of macroalgae. Syst. Appl. Microbiol. 10.1016/j.syapm.2013.11.004 24631661

[B13] Bondoso J. Balagué V. Gasol J. M. Lage O. M. (2013). Community composition of the Planctomycetes associated with different macroalgae. FEMS Microbiol. Ecol. 10.1111/1574-6941.12258 24266389

[B14] Burke C. Thomas T. Lewis M. Steinberg P. Kjelleberg S. (2011). Composition, uniqueness and variability of the epiphytic bacterial community of the green alga *Ulva australis*. ISME J. 5, 590–600 10.1038/ismej.2010.164 21048801 PMC3105733

[B15] Campbell A. H. Marzinelli E. M. Vergés A. Coleman M. A. Steinberg P. D. (2014). Towards restoration of missing underwater forests. PLoS ONE 9:e84106 10.1371/journal.pone.0084106 24416198 PMC3885527

[B16] Caracciolo A. B. Grenni P. Ciccoli R. Di Landa G. Cremisini C. (2005). Simazine biodegradation in soil: analysis of bacterial community structure by in situ hybridization. Pest Manag. Sci. 61, 863–869 10.1002/ps.1096 16015577

[B17] Cayrou C. Raoult D. Drancourt M. (2010). Broad-spectrum antibiotic resistance of *Planctomycetes* organisms determined by Etest. J. Antimicrob. Chemother. 65, 2119–2122 10.1093/jac/dkq290 20699245

[B18] Chouari R. Le Paslier D. Daegelen P. Ginestet P. Weissenbach J. Sghir A. (2003). Molecular evidence for novel planctomycete diversity in a municipal wastewater treatment plant. Appl. Environ. Microbiol. 69, 7354–7363 10.1128/AEM.69.12.7354-7363.2003 14660385 PMC309898

[B19] Costa R. Keller-Costa T. Gomes N. C. M. Da Rocha U. N. van Overbeek L. van Elsas J. D. (2013). Evidence for selective bacterial community structuring in the freshwater sponge *Ephydatia fluviatilis*. Microb. Ecol. 65, 232–244 10.1007/s00248-012-0102-2 22903086

[B20] De Oliveira L. S. Gregoracci G. B. Silva G. G. Salgado L. T. Filho G. A. Alves-Ferreira M. (2012). Transcriptomic analysis of the red seaweed *Laurencia dendroidea* (*Florideophyceae*, *Rhodophyta*) and its microbiome. BMC Genomics 13:487 10.1186/1471-2164-13-487 22985125 PMC3534612

[B21] DeLong E. F. Franks D. G. Alldredge L. (1993). Phylogenetic diversity of aggregate-attached vs. free-living marine bacterial assemblages. Limnol. Oceanogr. 38, 924–934 10.4319/lo.1993.38.5.0924

[B22] Devos D. P. Reynaud E. G. (2010). Intermediate steps. Science 330, 1187–1188 10.1126/science.1196720 21109658

[B23] Donadio S. Monciardini P. Sosio M. (2007). Polyketide synthases and nonribosomal peptide synthetases: the emerging view from bacterial genomics. Nat. Prod. Rep. 24, 1073–1109 10.1039/b514050c 17898898

[B24] Duque-Alarcón A. Santiago-Vázquez L. Z. Kerr R. G. (2012). A microbial community analysis of the octocoral Eunicea fusca. Electron. J. Biotechnol. 15, 1–9 10.2225/vol15-issue5-fulltext-11

[B25] Erbilgin O. McDonald K. L. Kerfeld C. A. (2014). Characterization of a planctomycetal organelle: a novel bacterial microcompartment for the aerobic degradation of plant saccharides. Appl. Environ. Microbiol. 80, 2193–2205 10.1128/AEM.03887-13 24487526 PMC3993161

[B26] Fuerst J. A. Gwilliam H. G. Lindsay M. Lichanska A. Belcher C. Vickers J. E. (1997). Isolation and molecular identification of planctomycete bacteria from postlarvae of the giant tiger prawn, *Penaeus monodon*. Appl. Environ. Microbiol. 63, 254–262 8979353 10.1128/aem.63.1.254-262.1997 PMC168317

[B27] Fuerst J. A. Sagulenko E. (2013). Nested bacterial boxes: nuclear and other intracellular compartments in plactomycetes. J. Mol. Microbiol. Biotechnol. 23, 95–103 10.1159/000346544 23615198

[B28] Fukunaga Y. Kurahashi M. Sakiyama Y. Ohuchi M. Tokota A. Harayama S. (2009). Phycisphaera mikurensis gen. nov., sp. nov., isolated from a marine alga, and proposal of phycisphaeraceae fam. nov., phycisphaerales ord. nov. and phycisphaerae classis nov. in the phylum planctomycetes. J. Gen. Appl. Microbiol. 55, 267–275 10.2323/jgam.55.267 19700920

[B29] Gade D. Stuhrmann T. Reinhardt R. Rabus R. (2005). Growth phase dependent regulation of protein composition in *Rhodopirellula baltica*. Environ. Microbiol. 7, 1074–1084 10.1111/j.1462-2920.2005.00784.x 16011746

[B30] Glockner F. O. Kube M. Bauer M. Teeling H. Lombardot T. Ludwig W. (2003). Complete genome sequence of the marine planctomycete *Pirellula* sp. strain 1. Proc. Natl. Acad. Sci. U. S. A. 100, 8298–8303 10.1073/pnas.1431443100 12835416 PMC166223

[B31] Goecke F. Thiel V. Wiese J. Labes A. Imhoff J. F. (2013). Algae as an important environment for bacteria—phylogenetic relationships among new bacterial species isolated from algae. Phycologia 52, 14–24 10.2216/12-24.1

[B32] Grube M. Köberl M. Lackner S. Berg C. Berg G. (2012). Host-parasite interaction and microbiome response: effects of fungal infections on the bacterial community of the Alpine lichen *Solorina crocea*. FEMS Microbiol. Ecol. 82, 472–481 10.1111/j.1574-6941.2012.01425.x 22671536

[B33] Guppy R. Bythell J. C. (2006). Environmental effects on bacterial diversity in the surface mucus layer of the reef coral *Montastraea faveolata*. Mar. Ecol. Prog. Ser. 328, 133–142 10.3354/meps328133

[B34] Halter D. Cordi A. Gribaldo S. Gallien S. Goulhen-Chollet F. Heinrich-Salmeron A. (2011). Taxonomic and functional prokaryote diversity in mildly arsenic-contaminated sediments. Res. Microbiol. 162, 878–887 10.1016/j.resmic.2011.06.001 21704701

[B35] Hempel M. Blume M. Blindow I. Gross E. M. (2008). Epiphytic bacterial community composition on two common submerged macrophytes in brackish water and freshwater. BMC Microbiol. 8:58 10.1186/1471-2180-8-58 18402668 PMC2386815

[B36] Hengst M. B. Andrade S. Gonzalez B. Correa J. A. (2010). Changes in epiphytic bacterial communities of intertidal seaweeds modulated by host, temporality, and copper enrichment. Microb. Ecol. 60, 282–290 10.1007/s00248-010-9647-0 20333374

[B37] Hollants J. Leliaert F. De Clerck O. Willems A. (2013). What we can learn from sushi: a review on seaweed-bacterial associations. FEMS Microbiol. Ecol. 83, 1–16 10.1111/j.1574-6941.2012.01446.x 22775757

[B38] Huang Y. Niu B. Gao Y. Fu L. Li W. (2010). CD-HIT Suite: a web server for clustering and comparing biological sequences. Bioinformatics 26, 680–682 10.1093/bioinformatics/btq003 20053844 PMC2828112

[B39] Ivanova A. O. Dedysh S. N. (2012). Abundance, diversity, and depth distribution of Planctomycetes in acidic northern wetlands. Front. Microbiol. 3:5 10.3389/fmicb.2012.00005 22279446 PMC3260447

[B40] Jensen S. I. Kühl M. Priemé A. (2007). Different bacterial communities associated with the roots and bulk sediment of the seagrass *Zostera marina*. FEMS Microbiol. Ecol. 62, 108–117 10.1111/j.1574-6941.2007.00373.x 17825072

[B41] Jeske O. Jogler M. Petersen J. Sikorski J. Jogler C. (2013). From genome mining to phenotypic microarrays: Planctomycetes as source for novel bioactive molecules. Antonie Van Leeuwenhoek 104, 551–567 10.1007/s10482-013-0007-1 23982431

[B42] Kulichevskaia I. S. Pankratov T. A. Dedysh S. N. (2006). Detection of representatives of the Planctomycetes in *Sphagnum* peat bogs by molecular and cultivation methods. Mikrobiologiia. 75, 389–396 16871807

[B43] Lachnit T. Blümel M. Imhoff J. F. Wahl M. (2009). Specific epibacterial communities on macroalgae: phylogeny matters more than habitat. Aquatic Biology 5, 181–186 10.3354/ab00149

[B44] Lachnit T. Meske D. Wahl M. Harder T. Schmitz R. (2011). Epibacterial community patterns on marine macroalgae are host-specific but temporally variable. Environ. Microbiol. 13, 655–665 10.1111/j.1462-2920.2010.02371.x 21078035

[B45] Lage O. (2013). Characterization of a planctomycete associated with the marine dinoflagellate *Prorocentrum micans* Her. Antonie Van Leeuwenhoek 104, 499–508 10.1007/s10482-013-9991-4 23929089

[B46] Lage O. Bondoso J. Lobo-Da-Cunha A. (2013). Insights into the ultrastructural morphology of novel Planctomycetes. Antonie van Leeuwenhoek, 1–10 10.1007/s10482-013-9969-2 23857394

[B47] Lage O. M. Bondoso J. (2011). *Planctomycetes* diversity associated with macroalgae. FEMS Microbiol. Ecol. 78, 366–375 10.1111/j.1574-6941.2011.01168.x 21726244

[B48] Liu M. Dong Y. Zhao Y. Zhang G. Zhang W. Xiao T. (2010). Structures of bacterial communities on the surface of *Ulva prolifera* and in seawaters in an *Ulva* blooming region in Jiaozhou Bay, China. World J. Microbiol. Biotechnol. 27, 1703–1712 10.1007/s11274-010-0627-9

[B49] Liu Y. Yao T. Jiao N. Kang S. Zeng Y. Huang S. (2006). Microbial community structure in moraine lakes and glacial meltwaters, Mount Everest. FEMS Microbiol. Lett. 265, 98–105 10.1111/j.1574-6968.2006.00477.x 17107422

[B50] Longford S. R. Tujula N. A. Crocetti G. R. Holmes A. J. Holmström C. Kjelleberg S. (2007). Comparisons of diversity of bacterial communities associated with three sessile marine eukaryotes. Aquat. Microb. Ecol. 48, 217–229 10.3354/ame048217

[B51] Lonhienne T. G. Sagulenko E. Webb R. I. Lee K. C. Franke J. Devos D. P. (2010). Endocytosis-like protein uptake in the bacterium *Gemmata obscuriglobus*. Proc. Natl. Acad. Sci. U.S.A. 107, 12883–12888 10.1073/pnas.1001085107 20566852 PMC2919973

[B52] Lucheta A. R. Otero X. L. Macías F. Lambais M. R. (2013). Bacterial and archaeal communities in the acid pit lake sediments of a chalcopyrite mine. Extremophiles 17, 941–951 10.1007/s00792-013-0576-y 23963670

[B53] Matsuo Y. Imagawa H. Nishizawa M. Shizuri Y. (2005). Isolation of an algal morphogenesis inducer from a marine bacterium. Science 307, 1598 10.1126/science.1105486 15761147

[B54] Meusnier I. Olsen J. L. Stam W. T. Destombe C. Valero M. (2001). Phylogenetic analyses of *Caulerpa taxifolia* (*Chlorophyta*) and of its associated bacterial microflora provide clues to the origin of the Mediterranean introduction. Mol. Ecol. 10, 931–946 10.1046/j.1365-294X.2001.01245.x 11348502

[B55] Miranda L. N. Hutchison K. Grossman A. R. Brawley S. H. (2013). Diversity and abundance of the bacterial community of the red macroalga *Porphyra umbilicalis*: did bacterial farmers produce macroalgae? PLoS ONE 8:e58269 10.1371/journal.pone.0058269 23526971 PMC3603978

[B56] Newsham K. K. Pearce D. A. Bridge P. D. (2010). Minimal influence of water and nutrient content on the bacterial community composition of a maritime Antarctic soil. Microbiol. Res. 165, 523–530 10.1016/j.micres.2009.11.005 20006478

[B57] Oliveira F.a.S. Colares G. B. Hissa D. C. Angelim A. L. Melo V. M. M. Lotufo T.M.C. (2013). Microbial epibionts of the colonial ascidians *Didemnum galacteum* and *Cystodytes* sp. Symbiosis 59, 57–63 10.1007/s13199-012-0210-2

[B58] Pilhofer M. Rappl K. Eckl C. Bauer A. P. Ludwig W. Schleifer K. H. (2008). Characterization and evolution of cell division and cell wall synthesis genes in the bacterial phyla *Verrucomicrobia*, *Lentisphaerae*, *Chlamydiae*, and *Planctomycetes* and phylogenetic comparison with rRNA genes. J. Bacteriol. 190, 3192–3202 10.1128/JB.01797-07 18310338 PMC2347405

[B59] Pimentel-Elardo S. Wehrl M. Friedrich A. B. Jensen P. R. Hentschel U. (2003). Isolation of planctomycetes from *Aplysina* sponges. Aquat. Microb. Ecol. 33, 239–245 10.3354/ame03323917545322

[B60] Piquet A. M. T. Bolhuis H. Davidson A. T. Buma A. G. J. (2010). Seasonal succession and UV sensitivity of marine bacterioplankton at an Antarctic coastal site. FEMS Microbiol. Ecol. 73, 68–82 10.1111/j.1574-6941.2010.00882.x 20455939

[B61] Reed D. W. Fujita Y. Delwiche M. E. Blackwelder D. B. Sheridan P. P. Uchida T. (2002). Microbial communities from methane hydrate-bearing deep marine sediments in a forearc basin. Appl. Environ. Microbiol. 68, 3759–3770 10.1128/AEM.68.8.3759-3770.2002 12147470 PMC124055

[B62] Reynaud E. G. Devos D. P. (2011). Transitional forms between the three domains of life and evolutionary implications. Proc. Biol. Sci. 278, 3321–3328 10.1098/rspb.2011.1581 21920985 PMC3177640

[B63] Rusch D. B. Halpern A. L. Sutton G. Heidelberg K. B. Williamson S. Yooseph S. (2007). The sorcerer II global ocean sampling expedition: northwest atlantic through eastern tropical pacific. PLoS Biol. 5:e77 10.1371/journal.pbio.0050077 17355176 PMC1821060

[B64] Santarella-Mellwig R. Franke J. Jaedicke A. Gorjanacz M. Bauer U. Budd A. (2010). The compartmentalized Bacteria of the *Planctomycetes-Verrucomicrobia-Chlamydiae* superphylum have membrane coat-like proteins. PLoS Biol. 8:e1000281 10.1371/journal.pbio.1000281 20087413 PMC2799638

[B65] Santarella-Mellwig R. Pruggnaller S. Roos N. Mattaj I. W. Devos D. P. (2013). Three-dimensional reconstruction of bacteria with a complex endomembrane system. PLoS Biol. 11:e1001565 10.1371/journal.pbio.1001565 23700385 PMC3660258

[B66] Schlesner H. (1994). The development of media suitable for the microorganisms morphologically resembling *Planctomyces* spp., *Pirellula* spp., and other *Planctomycetales* from various aquatic habitats using dilute media. Syst. Appl. Microbiol. 17, 135–145 10.1016/S0723-2020(11)80042-1

[B67] Schneider D. Arp G. Reimer A. Reitner J. Daniel R. (2013). Phylogenetic analysis of a microbialite-forming microbial mat from a hypersaline lake of the kiritimati atoll, central pacific. PLoS ONE 8:e66662 10.1371/journal.pone.0066662 23762495 PMC3677903

[B68] Stackebrandt E. (2002). Report of the ad hoc committee for the re-evaluation of the species definition in bacteriology. Int. J. Syst. Evol. Microbiol. 52, 1043–1047 10.1099/ijs.0.02360-0 12054223

[B69] Tekere M. Lötter A. Olivier J. Jonker N. Venter S. (2011). Metagenomic analysis of bacterial diversity of siloam hot water spring, limpopo, South Africa. Afr. J. Biotechnol. 10, 18005–18012 10.5897/AJB11.899

[B70] Van Teeseling M. C. F. Neumann S. Van Niftrik L. (2013). The anammoxosome organelle is crucial for the energy metabolism of anaerobic ammonium oxidizing bacteria. J. Mol. Microbiol. Biotechnol. 23, 104–117 10.1159/000346547 23615199

[B71] Wahl M. (2008). Ecological lever and interface ecology: epibiosis modulates the interactions between host and environment. Biofouling 24, 427–438 10.1080/08927010802339772 18686057

[B72] Ward N. Staley J. T. Fuerst J. A. Giovannoni S. Schlesner H. Stackebrandt E. (2006). The order Planctomycetales, including the genera Planctomyces, Pirellula, Gemmata and Isosphaera and the Candidatus genera Brocadia, Kuenenia and Scalindua, in The Prokaryotes: A Handbook on the Biology of Bacteria, Vol. 7, eds Dworkin M. Falkow S. Rosenberg E. Schleifer K. H. Stackebrandt E. (New York, NY: Springer), 757–793

[B73] Webster N. S. Bourne D. (2007). Bacterial community structure associated with the Antarctic soft coral, *Alcyonium antarcticum*. FEMS Microbiol. Ecol. 59, 81–94 10.1111/j.1574-6941.2006.00195.x 17233746

[B74] Webster N. S. Wilson K. J. Blackall L. L. Hill R. T. (2001). Phylogenetic diversity of bacteria associated with the marine sponge *Rhopaloeides odorabile*. Appl. Environ. Microbiol. 67, 434–444 10.1128/AEM.67.1.434-444.2001 11133476 PMC92596

[B75] Wecker P. Klockow C. Ellrott A. Quast C. Langhammer P. Harder J. (2009). Transcriptional response of the model Planctomycete *Rhodopirellula baltica* SH1^(*T*)^ to changing environmental conditions. BMC Genomics 10:410 10.1186/1471-2164-10-410 19725962 PMC2754498

[B76] Wegner C. E. Richter-Heitmann T. Klindworth A. Klockow C. Richter M. Achstetter T. (2013). Expression of sulfatases in *Rhodopirellula baltica* and the diversity of sulfatases in the genus *Rhodopirellula*. Mar. Genomics 9, 51–61 10.1016/j.margen.2012.12.001 23273849

[B77] Winkelmann N. Harder J. (2009). An improved isolation method for attached-living *Planctomycetes* of the genus *Rhodopirellula*. J. Microbiol. Methods 77, 276–284 10.1016/j.mimet.2009.03.002 19303037

[B78] Yakimov M. M. Cappello S. Crisafi E. Tursi A. Savini A. Corselli C. (2006). Phylogenetic survey of metabolically active microbial communities associated with the deep-sea coral Lophelia pertusa from the Apulian plateau, Central Mediterranean Sea. Deep-Sea Res. I 53, 62–75 10.1016/j.dsr.2005.07.005

[B79] Yoon J. Jang J. H. Kasai H. (2014). Algisphaera agarilytica gen. nov., sp. nov., a novel representative of the class Phycisphaerae within the phylum Planctomycetes isolated from a marine alga. Antonie Van Leeuwenhoek 105, 317–324 10.1007/s10482-013-0076-1 24264814

[B80] Zeng Y. X. Yan M. Yu Y. Li H. R. He J. F. Sun K. (2013). Diversity of bacteria in surface ice of Austre Lovénbreen glacier, Svalbard. Arch. Microbiol. 195, 313–322 10.1007/s00203-013-0880-z 23474777

[B81] Zhang W. Wu X. Liu G. Chen T. Zhang G. Dong Z. (2013). Pyrosequencing reveals bacterial diversity in the rhizosphere of three phragmites australis ecotypes. Geomicrobiol. J. 30, 593–599 10.1080/01490451.2012.740145

[B82] Zhao Z. Luo K. Chen G. Yang Y. Mao Z. Liu E. (2010). Analysis of bacterial diversity in rhizosphere of cucumber in greenhouse by the methods of metagenomic end-random sequencing and 16S rDNA technology. Shengtai Xuebao/ Acta Ecologica Sinica 30, 3849–3857

[B83] Zhu P. Li Q. Wang G. (2008). Unique microbial signatures of the alien hawaiian marine sponge *Suberites zeteki*. Microb. Ecol. 55, 406–414 10.1007/s00248-007-9285-3 17676375

